# Comparing Fusion Rates Between Fresh-Frozen and Freeze-Dried Allografts in Anterior Cervical Discectomy and Fusion

**DOI:** 10.1016/j.wnsx.2022.100126

**Published:** 2022-05-31

**Authors:** Derron Yu, Paramjyot Singh Panesar, Connor Delman, Benjamin W. Van, Machelle D. Wilson, Hai Van Le, Rolando Roberto, Yashar Javidan, Eric O. Klineberg

**Affiliations:** 1 Spine Center, University of California, Davis, Sacramento, California, USA; 2 University of California, Davis School of Medicine, Sacramento, California, USA; 3 Department of Orthopaedics, University of California, Davis, Sacramento, California, USA; 4 University of California, Davis Clinical and Translational Science Center, Sacramento, California, USA

**Keywords:** ACDF, Allografts, Cervical radiculopathy, Freeze-dried allografts, Fresh-frozen, Fusion rates, Spine, ACDF, Anterior cervical discectomy and fusion, AP/Lat, Anterior-posterior/lateral, BMP, Bone morphogenic protein, CT, Computed tomography, NDI, Neck Disability Index, NSAID, Nonsteroidal antiinflammatory drug

## Abstract

**Objective:**

The objective of this retrospective study is to compare the fusion rates in anterior cervical discectomy and fusion surgery using freeze-dried versus fresh-frozen allografts.

**Methods:**

The study comprised 79 patients. Fifty-one patients received freeze-dried allograft (106 total spinal levels) and 28 patients received fresh-frozen allograft (50 total spinal levels). Fusion was assessed through trabecular bridging on follow-up anterior-posterior/lateral radiographs. Trabecular bridging was assessed on the superior and inferior borders of each spinal level and given a fusion grade. Complete fusion is defined as >50% bridging between superior and inferior borders of the bone graft; union is complete fusion in <26 weeks; delayed union is complete fusion after 26 weeks; and fibrous union is <50% bridging at ≥1 borders over 52 weeks.

**Results:**

All spinal levels reached complete fusion for both graft types. Of the freeze-dried treated cervical spinal levels, 77.35% (82/106) reached union (adequate trabecular bridging within 6 months) without delay compared with 80% (35/50) for the fresh-frozen bone graft group (*P* = 0.85). There was no significant difference in time-to-fusion analysis and no significant association between delayed union and any patient factors. In assessing Neck Disability Index (NDI), freeze-dried allografts did show a significantly greater decrease in NDI scores at 6 months (*P* = 0.03). At the 1 year follow-up, improvements in NDI were consistent in both allografts (*P* = 0.9647).

**Conclusions:**

From this study, freeze-dried and fresh-frozen allografts showed comparable rates of union, and both allografts can be used interchangeably for anterior cervical discectomy and fusion.

## Introduction

Anterior cervical discectomy and fusion (ACDF) is used to treat a variety of cervical diseases, including degenerative disease, myelopathy, radiculopathy, and traumatic injuries.^1^ ACDF has become one of the most common cervical spine procedures in the United States. The procedure involves addressing the underlying disease, removing the degenerated vertebral disc, and replacing it with bone graft to promote fusion. One complication of this procedure is pseudarthrosis, or nonunion, which is defined as a failure of fusion between cervical levels. If pseudarthrosis does occur, a revision surgery is often required to achieve a successful fusion. Pseudarthrosis is a leading cause of pain postoperatively, accounting for 45%–56% of revision surgery.^1^ Given the morbidity associated with nonunion after ACDF, a more complete understanding and analysis of the available graft options are necessary to facilitate preoperative planning and improve surgical outcomes.

In ACDF, the gold standard for grafts is an autograft from the patient’s iliac crest.^2^ Autografts have no immune response, less infection risk, and more inherent growth factors to help with graft incorporation.^2^, ^3^, ^4^ However, the potential negative sequelae of iliac autograft include donor site morbidity such as pain, infection, hematoma, fracture, and wound healing complications.^5^, ^6^, ^7^ These complications can add cost as a result of reoperation and prolonged postoperative disability. Because of these donor site morbidities, surgeons have begun to look to allografts as an alternative.

Allografts are often harvested from the anterior iliac crest, fibula, or femur of donors.^3^^,^^5^ These grafts eliminate donor site morbidity and have been shown to have similar rates of fusion compared with autografts, specifically in single-level fusions.^2^^,^^7^, ^8^, ^9^, ^10^, ^11^, ^12^ Allografts have osteoinductive and osteoconductive properties but have lost osteogenic capacity through processing and sterilization.^3^ Allografts must be prepared and processed to reduce the risk of immunologic mismatch and inflammatory reactions, which can impede the rate of fusion. Fresh-frozen allografts are put through an antibiotic wash and cooled at –70°C.^3^^,^^4^ The allografts then can remain in –20°C to maintain a shelf life of 5 years.^3^ Freeze-dried allografts go through additional steps of lyophilization, an extraction of its water content to 5%, and irradiation.^3^^,^^4^^,^^13^ These procedures allow the eradication of viral DNA in the freeze-dried allografts but lead to denaturation of bone morphogenic proteins (BMP), which are essential in osteoinduction.^3^^,^^4^^,^^13^ After this process, freeze-dried grafts can remain at room temperature, allowing more convenience, with a shelf life of 5 years.^3^ This strategy can benefit hospital inventory management and storage. Compared with fresh-frozen allografts, freeze-dried allografts are more brittle and have decreased compressive strength because of the more rigorous processing methods.^3^^,^^13^^,^^14^ However, freeze-dried allografts, through irradiation, are less likely to invoke an immune response, which is important for successful graft fusion.^4^^,^^14^ Disease transmission through bone grafts, such as bacterial infections, requires resection of the bone graft and treatment of the infection. With the current protocols of processing allografts, the risk of transmitting viral and bacterial diseases has decreased drastically. Bacterial transmission in nonmassive allografts, such as morselized bone grafts, are at 0.7%.^15^ The risk of transmission for viral diseases is also low.^14^ Human immunodeficiency virus has a risk of 1 in 1.6 million in properly screened bone allografts and there have been only 2 reported incidences since 1985.^14^

In this study, we compare the fusion rates between fresh-frozen and freeze-dried allografts using radiographic analysis. We also take into consideration patients’ medical comorbidities and the resultant effect on fusion rates. We hypothesize that fresh-frozen allografts, given their preserved structural integrity and osteogenic proteins, reduce the rate of pseudarthrosis in patients and possibly achieve fusion sooner.

## Methods

This was a retrospective study of patients who underwent ACDF with either a fresh-frozen bone graft or a freeze-dried bone graft to treat their underlying myelopathy, radiculopathy, or instability. This is a single-surgeon single-center study by the senior author at the University of California–Davis Medical Center over an 8-year span, from July 2014 to June 2020. Bone graft selection was based on availability for procedure and surgeon preference over time. From 2014 to 2017, the medical center mainly had freeze-dried allografts available for ACDF. From 2017 to 2020, predominantly fresh-frozen allograft were available for the procedure. Comorbidities and patient demographics that may affect fusion such as smoking history, diabetes, nonsteroidal antiinflammatory drug (NSAID) use, and sex were recorded. These factors have shown to affect the rate of fusion and pseudarthrosis in ACDF and other spinal procedures.^1^^,^^16^^,^^17^

### Bone Graft Preparation

Freeze-dried allografts were preserved using lyophilization to decrease the water content to 6%. It is then rehydrated and maintained in a sterile environment before the procedure. The fresh-frozen allografts were preserved and stored at –40C to –90C. Before the procedure, the graft is thawed in a sterile irrigant and rinsed. All allografts have been subject to aseptic processing conditions in compliance with the ISO (International Organization for Standardization) class 4 environment. All donor samples are tested and infectious disease tests were negative.

After ACDF, patients were placed in a hard cervical collar for 4–12 weeks depending on the number of levels fused and interval healing.

**Figure 1:**
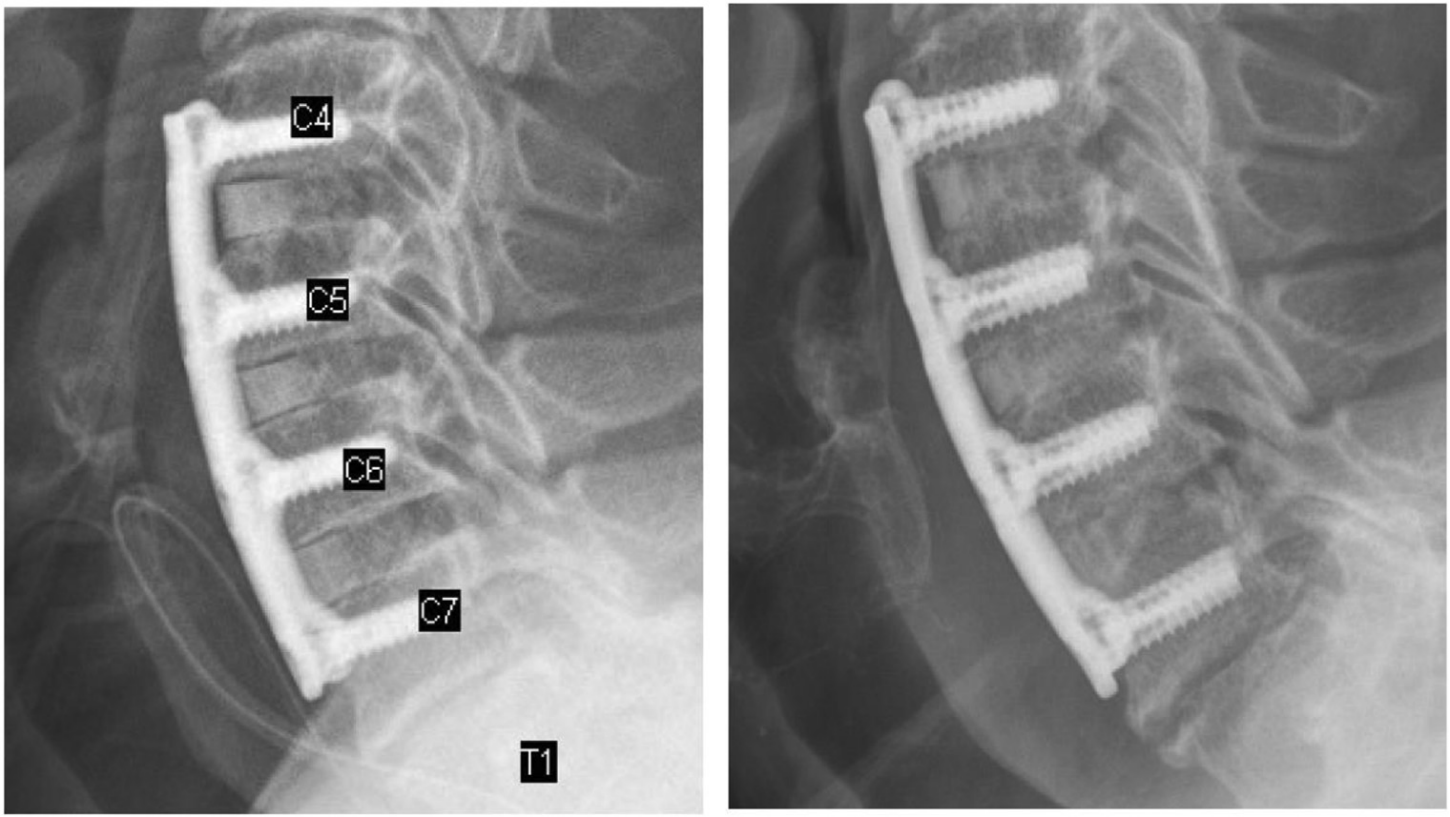
Adequate trabecular bridging. The imaging on the *left* shows spinal levels immediately postoperatively (1 day). Immediate postoperative imaging shows radiolucent areas between spinal levels indicating inadequate fusion and nonfusion. The imaging on the *right* was taken in the same patient at their 6 months follow-up. At 6 months follow-up, spinal levels show decreased radiolucency and increased trabecular bridging in >50% of the superior and inferior borders. This is an example of adequate fusion and because it was achieved at the 6-month time mark, it is graded as a union. Achieving fusion after the 6-month mark is considered a delayed union. Being unable to achieve fusion at 52 weeks is considered fibrous union.

For postoperative follow-up, periodic radiographs were obtained from various time points for each patient, ranging from 3 months to 12 months. Seventy-nine patients had anterior-posterior/lateral (AP/Lat) radiographs. Radiographs were analyzed by 1 orthopedic resident and 2 medical students affiliated with the University of California–Davis Medical Center. Each evaluator analyzed all the radiographs and an average of the 3 evaluators’ percentage of trabecular bridging was used. The intraclass correlation coefficient among raters was 0.98, indicating a high interrater reliability. For the AP/Lat radiographs, fusion was graded based on trabecular bridging on the superior and inferior border for each fusion level. Trabecular bridging was given a percentage to correspond to the extent of fusion. Complete fusion is defined as >50% bridging between superior and inferior borders. Fusion grades were then categorized into 3 tiers: union, complete fusion within 26 weeks; delayed union, complete fusion after 26 weeks; and fibrous union, <50% bridging at ≥1 borders over 52 weeks^11^^,^^18^ (Figure 1). Furthermore, patient Neck Disability Index (NDI) was recorded to assess differences in postoperative discomfort and pain for patients.

#### Statistical Analysis

Hierarchical logistic regression models were fit to test for associations between delayed union and various risk factors. Interactions between the risk factor and graft type were included in the models. Kaplan-Meier curves were fit and the log-rank test conducted, to test for differences between graft type and time to fusion. In time-to-fusion analysis, patient radiographs were obtained at 3-month, 6-month, and 12-month postoperative intervals. Difference-in-differences analyses were conducted using linear models to examine the effect of graft type on change in NDI, controlling for baseline NDI. All analyses were conducted using SAS version 9.4 (SAS Institute Inc., Cary, North Carolina, USA).

#### Ethics

This study was approved by the institutional review board under approval number 1574467-1. All patients were fully legally competent and informed consent was obtained for use in research. There were no conflict of interests and no funding was received for this study.

## Results

A total of 79 patients met the inclusion criteria and were eligible for evaluation. The freeze-dried allograft group comprised 51 patients (23 men and 28 women). In these patients, freeze-dried allografts were used for 106 cervical spinal levels. The fresh-frozen allografts group comprised 28 patients (16 men and 12 women). Fresh-frozen allografts were used in 50 cervical spinal levels. Postoperative complications for patients involved radiculopathy and myelopathy, which improved during recovery. One patient from each allograft group had dysphagia during recovery but did not pursue further treatment for it. There were no noted revision procedures or viral infections after the procedure. Further demographics are shown in Table 1.

**Table 1:** Comparison of Patient Factors, Such as Sex, Smoking History, Nonsteroidal Antiinflammatory Drug Use, Diabetes, History of Osteoporosis, Hyperthyroidism, Levels Fused, Age, and Postoperative Complications

Factor	Patients Receiving Fresh-Frozen Bone Grafts (N = 27)	Patients Receiving Freeze-Dried Bone Grafts (N = 51)	*P* Value
Female sex	12 (44.44)	29 (56.86)	0.30
Male sex	15 (55.56)	22 (43.14)	0.29
Smoker	13 (48)	28 (55)	0.57
Nonsteroidal antiinflammatory drugs	2 (7.41)	5 (9.80)	0.72
Diabetes	5 (18.52)	11 (21.57)	0.75
Osteoporosis	1 (3.70)	2 (3.92)	0.96
Hyperthyroidism	0 (0)	1 (1.96)	0.46
1-level fusion	10 (37.04)	14 (27.45)	0.38
2-level fusion	11 (40.74)	20 (39.22)	0.89
3-level fusion	6 (22.22)	17 (33.33)	0.30
Age (years), mean (standard deviation)	60.3 (9.7)	58.1 (12.3)	0.4
Postoperative complications	5 (18.52)	4 (7.84)	0.16

Values are number (%) except where indicated otherwise. *P* values showed no significant changes or differences between patients who received fresh-frozen or freeze-dried bone grafts.

All spinal levels reached complete fusion for both graft types. Of the freeze-dried treated cervical spinal levels, 77.35% (82/106) reached union (complete fusion within 6 months) without delay compared with 80% (35/50) for the fresh-frozen bone graft group (*P* = 0.85) (Table 2). There were no instances of any fibrous union from either bone grafts.

**Table 2:** Odds Ratio of Union (Adequate Fusion in 6 Months) in Patient Factors That May Contribute to Rate of Union

Risk Factor	Unadjusted Odds Ratio	*P* Value	Interaction *P* Value
Graft type (freeze-dried vs. fresh-frozen)	0.89 (0.25–3.13)	0.85	—
Male sex	0.41 (0.12–1.4)	0.15	0.95
History of smoking	1.3 (0.40–4.3)	0.67	0.26
Current smoking	1.3 (0.15–11.1)	0.82	0.95
NSAIDs	1.6 (0.24–10.6)	0.63	0.75
Diabetes	0.95 (0.23–4.0)	0.95	Did not converge

*P* values mostly show no significant changes in odds of union in various patient factors. Interaction *P* value shows whether the effect of patient factors (sex, smoking, NSAID, and diabetes) on union differed between patients receiving freeze-dried or fresh-frozen allografts.

NSAID, nonsteroidal antiinflammatory drug.

**Figure 2:**
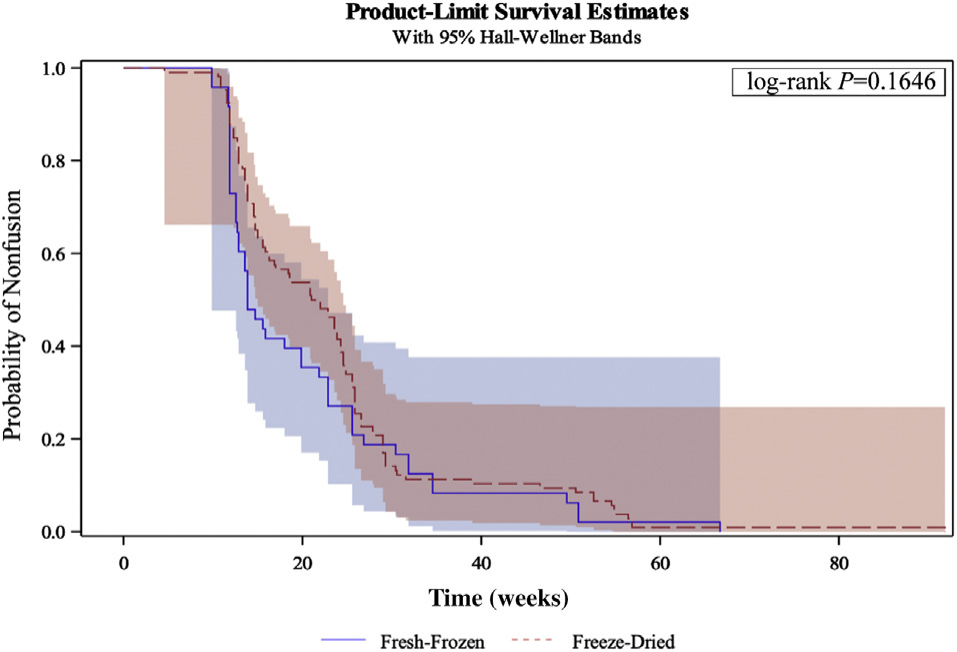
Kaplan-Meier curve analysis of time to fusion. Each patient’s time to fusion (adequate trabecular bridging on superior and inferior borders) was analyzed to observe whether freeze-dried or fresh-frozen allografts reached fusion sooner. Freeze-dried allografts (*red dotted line*) nor fresh-frozen allografts (*solid blue line*) did not show a significant difference in their time to fusion (log-rank *P* = 0.1646). Fresh-frozen decrease in Neck Disability Index, –6.588. Freeze-dried decrease in Neck Disability Index, –12.292. *P* = 0.03.

Patient radiographs were obtained at the 3-month, 6-month, and 12-month intervals to assess time-to-fusion analysis. There was no statistically significant difference between the graft type in time to fusion (*P* = 0.1646) (Figure 2).

For the univariate logistic regression models, there was no significant association between delayed union and any patient factors (Table 2). For the models testing for interactions between graft type and the risk factors, there were also no significant associations or interactions. The diabetes model did not converge because there were no diabetic patients with the fresh-frozen graft type (see Table 2).

**Figure 3:**
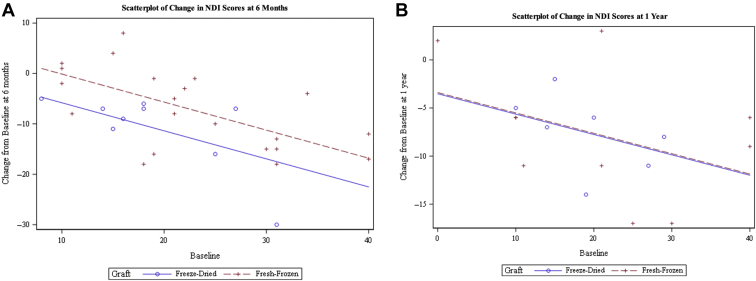
(**A**) Scatterplot with regression lines of Neck Disability Index (NDI) at 6 months postoperatively. NDI was recorded from patients at 6 months postoperatively. Baseline (preoperative) NDI was also recorded and controlled for. Patients with freeze-dried allografts are shown by *blue circles with a solid blue line*. Patients with fresh-frozen allografts are shown by *red crossed with dotted red lines*. Patients with freeze-dried bone grafts showed a statistically greater decrease in NDI in any given baseline score compared with patients with fresh-frozen bone grafts (*P* = 0.03). Fresh-frozen decrease in NDI score: –7.7. Freeze-dried decrease in NDI score: –7.8. *P* = 0.9647. (**B**) Scatterplot with regression lines of NDI at 1 year postoperatively. NDI was recorded from patients at 1 year postoperatively. Baseline (preoperative) NDI was also recorded and controlled for. Patients with freeze-dried allografts are shown by *blue circles with a solid blue line*. Patients with fresh-frozen allografts are shown by *red crossed with dotted red lines*. There were no statistical difference in changes of NDI between patients who received freeze-dried or fresh-frozen bone grafts for any given baseline score (*P* = 0.9647).

In our study, 38 patients had recorded an NDI at preoperative baseline, 6 months follow-up, and 1 year follow-up (Figure 3A and B). For the difference-in-differences analysis of NDI, there were significant differences at 6 months (*P* = 0.03) but not at 1 year (*P* = 0.96). After controlling for baseline, patients who received freeze-dried allografts had on average a decrease of 12.292 in NDI, whereas the patients who received fresh-frozen allografts had a decrease of 6.588 at the 6-month interval compared with preoperative NDI. Freeze-dried allografts showed a greater decrease of 5.7 in NDI score (*P* = 0.03) compared with fresh-frozen allografts at the 6-month interval. At 1 year follow-up, NDI decreases were not statistically significant. Freeze-dried allografts had a decrease of –7.7 and fresh-frozen allografts had a decrease in 7.8 in NDI compared with preoperative NDI. Freeze-dried bone grafts had a nonsignificant greater decrease of 0.122 NDI points (*P* = 0.9647) (see Figure 2). Of these 38 patients, 5 patients were graded as having delayed union, 2 with freeze-dried bone grafts, and 3 with fresh-frozen bone grafts. The 2 patients with freeze-dried bone allografts with delayed union had a change in NDI of –12 and –5 at the 6-month interval, with no additional change to NDI score at the 12-month interval. In the fresh-frozen allografts, 1 patient had an increase of 8 points in NDI, 1 patient with a change of –1 NDI, and 1 patient with a change of –13 at the 6-month interval, with no additional change to NDI at the 12-month interval. At the 6-month interval, patients with freeze-dried allografts showed significantly greater improvements in NDI compared with patients with fresh-frozen allografts. At the 12-month interval, both patients with freeze-dried and patients with fresh-frozen allografts showed comparable improvements in NDI.

## Discussion

We found no significant difference in fusion rates for ACDF using freeze-dried allografts versus fresh-frozen allografts. All bone grafts achieved complete fusion by 1 year. We did find that fresh-frozen allografts did have a slightly higher rate of union by 6 months (77.35% freeze-dried vs. 80% fresh-frozen). This finding does correspond well with the existing literature showing that patients with fresh-frozen allografts have had improved fusion rates in other procedures such as anterior lumbar interbody fusion.^19^ This improvement may be a result of the preservation of osteoinductive factors, such as BMP, in the fresh-frozen allografts and the denaturation of these factors in the freeze-dried allografts.^3^^,^^4^^,^^13^ Interpretation of the results showed that both grafts had no differences in fusion and union in the short-term and long-term. Fresh-frozen bone grafts have gone through an antibiotic wash and a less protein-damaging processing method.^3^^,^^4^ This strategy allows these types of grafts to retain more proteins, such as BMPs, to help promote bone fusion and growth.^3^^,^^4^^,^^13^ Freeze-dried bone grafts have gone through additional steps of irradiation, lyophilization, and water extraction, which strip away the BMPs, decrease its compressive strength, and make freeze-dried bone grafts more brittle.^3^^,^^4^^,^^13^^,^^14^ This situation can explain the slightly improved rate of union in fresh-frozen bone grafts at 6 months. However, the improved rate is not significant, and there was no incident of pseudarthrosis between either grafts at 1 year.

There were no significant associations between patient factors (i.e., sex, history of smoking, NSAID use, and diabetes) and risk for delayed union. We specifically investigated these risk factors because of their known effects on fusion. NSAIDs, through various studies, have been shown to inhibit bone healing from fractures and spinal fusion procedures in both mice models and human studies.^20^ The mechanisms are unknown; however, there are a few theories such as the role of cyclooxygenase 2 in the promotion of differentiation of mesenchymal cells to osteoblasts, promotion of angiogenesis, and pain relief that promotes more weight-bearing activities on the affected bone and inhibits healing.^20^ Smoking can also negatively affect bone healing by interrupting blood supply to the bone and decreasing expression of cytokines and proteins such as BMPs and vascular endothelial growth factor. These effects inhibit angiogenesis and decrease the delivery of oxygen and nutrients to the healing site. However, the effect of smoking on the development of nonunion in ACDF and cervical procedures is unclear, with some studies showing no changes in fusion rate between smokers and nonsmokers.^12^^,^^21^ Diabetes is another patient factor that has shown negative effects in bone healing. In mice models, the metabolic dysregulation associated with diabetes has been found to negatively affect the quality and density of fused bone masses, but it does not affect the rate of fusion.^22^ Sex hormones, such as testosterone and estrogen, have been shown to play a role in bone growth and maintenance. This situation can be seen in postmenopausal, estrogen-deficient women and their increased risk of osteoporosis. In this study, 80.9% of our female patients (34/42) were postmenopausal but did not show a significant decrease in fusion rates. Our results are consistent with other studies that have also shown no significant changes in fusion rates in ACDF procedures between premenopausal and postmenopausal women.^23^^,^^24^

The lack of significant changes in fusion rates could be attributed to the location of the procedure and bone graft placement. Cervical procedures may lead to a more consistent fusion rates regardless of patient factors or bone graft type as a result of increased blood flow and decreased weight bearing compared with other locations on the spine such as the thoracic and lumbar regions.

When assessing changes in NDI in patients, there was a significant decrease in scores from their preoperative baseline for the freeze-dried bone grafts at 6 months follow-up. At the 1 year follow-up, both patients with freeze-dried and patients with fresh-frozen bone grafts achieved similar decreases in NDI scores compared with their preoperative baseline scores. These results could be explained by the difference in processing methods between the 2 bone grafts. Fresh-frozen bone grafts are typically minimally processed compared with freeze-dried bone grafts. This factor may cause fresh-frozen bone grafts to have more cellular debris and to be more antigenic.^14^ This situation can induce an immune response in the patient and may cause rejection of the graft or discomfort for the patient.^14^ Fresh-frozen bone grafts may increase patient discomfort in the short-term; however, in the long term, fresh-frozen grafts were comparable to freeze-dried bone grafts in union and improvement in NDI. We were unable to find a correlation with change in NDI and in union at 6 months or 1 year in either graft type. If pain in the early postoperative period is important in select patients, freeze-dried bone grafts may offer early advantages over fresh-frozen and should be considered.

There were a few limitations to this study. Because of its retrospective nature, the study could not ensure that every patient obtained follow-up radiographs at the same designated time point. Some patients’ first postoperative radiograph was obtained after the 6-month window, which makes the determination of a delayed union difficult. The study is also limited by the number of patients who were included. This limitation can make it difficult to analyze the effects of smoking and other comorbidities on fusion rates. Another limitation is the type of radiographs that were used to grade fusion. Most of the patients in this study had received AP/Lat radiographs, rather than computed tomography (CT) imaging or flexion/extension radiographs. CT imaging is considered the most accurate way to assess fusion; however, because of its cost and exposure to radiation, not many patients were subjected to CT imaging.^25^ The next best option in radiographs is measuring the spinous distances in flexion and extension radiographs, which has been shown to have similar accuracy to CT imaging.^22^, ^23^, ^24^ However, in this study, few patients had flexion/extension radiographs on follow-up appointments, making the fusion criteria on AP/Lat radiographs the only option. Grading and assessing fusion in spinal levels can also be subjective and vary between evaluators. However, interobserver reliabilities showed a high interrater reliability.

This study found that there is no difference in the rates of union between freeze-dried and fresh-frozen allografts in ACDF at 1 year. Both types of allografts were shown to achieve union in the long-term. There may be a slight increase in union rates in the short-term for fresh-frozen allografts; however, the results were not statistically significant. We were also unable to identify any patient factors that significantly influenced union at 1 year. Further power analysis show that 19,000 spinal levels are needed to determine a difference. Freeze-dried bone grafts showed significantly improved short-term (6 month) NDI postoperatively compared with fresh-frozen grafts with no difference at 1 year. The marked improvement of NDI at 6 months follow-up for patients with freeze-dried allografts may be a result of the proinflammatory and antigenic nature of fresh-frozen allografts. This study showed there is no statistical significance in rates of union and NDI improvements at 1 year follow-up between fresh-frozen and freeze-dried allografts used in ACDF procedures. Furthermore, the freeze-dried and fresh-frozen allografts used at the University of California–Davis Medical Center have the same cost, at $1150. This study suggests that either type of allograft can be used for ACDF based on availability at the facility without compromising rates of union and patient outcome scores in the long-term.

## CRediT authorship contribution statement

**Derron Yu:** Methodology, Investigation, Writing – original draft, Supervision, Project administration, Supervision, Data curation, Visualization. **Paramjyot Singh Panesar:** Investigation, Data curation. **Connor Delman:** Methodology, Investigation, Data curation. **Benjamin W. Van:** Investigation, Data curation. **Machelle D. Wilson:** Formal analysis. **Hai Van Le:** Writing – review & editing. **Rolando Roberto:** Writing – review & editing. **Yashar Javidan:** Writing – review & editing. **Eric O. Klineberg:** Supervision, Project administration, Conceptualization, Methodology, Visualization, Investigation, Resources.

## Conflict of interest statement

The authors declare that the article content was composed in the absence of any commercial or financial relationships that could be construed as a potential conflict of interest.
